# Prevalence and factors associated with chronic school absenteeism among 207,107 in-school adolescents: Findings from cross-sectional studies in 71 low-middle and high-income countries

**DOI:** 10.1371/journal.pone.0283046

**Published:** 2023-05-10

**Authors:** Md. Ashfikur Rahman, Andre M. N. Renzaho, Satyajit Kundu, Md. Abdul Awal, Md. Ashikuzzaman, Lijun Fan, Bright Opoku Ahinkorah, Joshua Okyere, Joseph Kihika Kamara, Rashidul Alam Mahumud

**Affiliations:** 1 Development Studies Discipline, Social Science School, Khulna University, Khulna, Bangladesh; 2 Professor of Humanitarian and Development Studies, School of Medicine and Health, Western Sydney University, Campbelltown, Australia; 3 Global Health Institute, North South University, Dhaka, Bangladesh; 4 Faculty of Nutrition and Food Science, Patuakhali Science and Technology University, Dumki, Patuakhali, Bangladesh; 5 Electronics and Communication Engineering Discipline, Khulna University, Khulna, Bangladesh; 6 Development Studies Discipline, Khulna University, Khulna, Bangladesh; 7 School of Public Health, Southeast University, Nanjing, China; 8 School of Public Health, Faculty of Health, University of Technology Sydney, Sydney, Australia; 9 Department of Population and Health, University of Cape Coast, Cape Coast, Ghana; 10 World Vision International, East Africa Regional Office, Karen, Nairobi, Kenya; 11 NHMRC Clinical Trials Centre, Faculty of Medicine and Health, The University of Sydney, Camperdown, New South Wales, Australia; Spreeha Foundation Bangladesh / The University of Sydney / The University of Southern Queensland, AUSTRALIA

## Abstract

**Background:**

Despite the negative impact of chronic school absenteeism on the psychological and physical health of adolescents, data on the burden of adolescent chronic school absenteeism (ACSA) and interventions and programs to address it are lacking. We estimated the global, regional and national level prevalence of ACSA and its correlation with violence and unintentional injury, psychosocial, protective, lifestyle, and food security-related factors among in-school adolescents across low and middle-income, and high-income countries (LMICs–HICs).

**Objectives:**

This study aimed to estimate the prevalence of chronic school absenteeism (CSA) as well as to determine its associated factors among in-school adolescents across 71 low-middle and high-income countries.

**Methods:**

We used data from the most recent Global School-based Student Health Survey of 207,107 in-school adolescents aged 11–17 years in 71 LMICs-HICs countries across six WHO regions. We estimated the weighted prevalence of ACSA from national, regional and global perspectives. Multiple binary logistic regression analyses were used to estimate the adjusted effect of independent factors on ACSA.

**Results:**

The overall population-weighted prevalence of CSA was 11·43% (95% confidence interval, CI: 11·29–11·57). Higher likelihood of CSA was associated with severe food insecurity, peer victimisation, loneliness, high level of anxiety, physically attack, physical fighting, serious injury, poor peer support, not having close friends, lack of parental support, being obese, and high levels of sedentary behaviours. Lower likelihood of CSA was associated with being female (odds ratio, OR = 0·76, 95% CI: 0·74–0·78).

**Conclusion:**

Our findings indicate that a combination of different socio-economic factors, peer conflict and injury factors, factors exacerbate CSA among adolescents. Interventions should be designed to focus on these risk factors and should consider the diverse cultural and socioeconomic contexts.

## Introduction

Education is widely regarded as vital for promoting human rights, better health, and economic progress around the world. Early age represents a significant window to address inequities in school attendance. Consequently, the right to education has been enshrined into national and international instruments. The Sustainable Development Goal (SDG) 4 (ensure inclusive and equitable quality education and encourage lifelong learning opportunities for everyone by 2030), has provided an opportunity to monitor progress made in education [1]. Despite these initiatives, school absenteeism remains a serious concern for most low-and middle-income countries (LMICs), with many adolescents around the world skipping school [2, 3]. Students who experience chronic school absenteeism (CSA) that is, they miss at least 18 days or 10% a year in school [4, 5]—are seriously vulnerable to falling behind in school [6, 7]. There are several country-specific studies on truancy [8–12], (students who missed classes or school without permission in the last 30 days) but there is a dearth of literature on CSA at international and national levels. This makes global comparisons of the prevalence of CSA and related behaviours among adolescents difficult. The prevalence of adolescent chronic school absenteeism (CSA) varies by country and socio-economic contexts, and remains a latent crisis [13].

CSA is associated with a high degree of school dropout, poor psychological wellbeing and physical health, poor academic performance as well as social development [14]. Additionally, CSA exacerbates the risk of non-participation in the workforce, and subsequent long-term poverty [7, 15–17]. CSA is influenced by a combination of individual, family, and social factors [17]. Previous studies have shown that when parents become involved in the academic activities of their children (i.e., attending parents-teacher meetings, monitoring homework, etc.,), the risk of CSA reduces [18]. This implies that the absence of such parental support is likely to result in adolescents becoming indifferent towards schooling, thereby translating into CSA. Also, adolescents yearn to belong to groups where they have similar characteristics; hence, they form strong ties with their peers [19]. Therefore, it is postulated that adolescents who are unable to have a sense of belonging would resort to CSA as a conduit to belong [20].

To date, there is no global or regional evidence to show how violence and unintentional injury, psychosocial, protective, lifestyle, and food security-related factors as well as parental, peer and social support predispose adolescents to CSA. This gap in the current discourse and scholarship on CSA warrants the need for a large-scale comprehensive population-based study on CSA among adolescents to explore the nuances and associated factors at the global, regional and national levels. We aimed to estimate the prevalence of CSA and its correlation with violence and unintentional injury, psychosocial, protective, lifestyle, and food security-related factors among school-going adolescents across LMICs and high-income countries (HICs). This study provides an opportunity to understand the extent to which support systems affect CSA in the context of country income groups.

## Methods

### Data sources

This study is based on secondary data from the most recent Global School-based Student Health Survey (GSHS). The data was collected between 2003 and 2015. The GSHS a low-cost school-based survey that collects data on different aspects of adolescents’ health behaviours and on protective factors associated with morbidity and mortality worldwide [21, 22]. The survey tools and methods in each GSHS were tailored to each country context, but this study design and participant selection procedures were similar across the GSHS countries. GSHS employs two-stage sampling design was used where the first level of GSHS sample selection process was schools and the class rooms were considered as the second level. Cross-sectional design to collect data using self-administered questionnaire during one regular class period in the schools. The GSHS uses a standardized scientific sample selection process; common school-based methodology. The comprehensive survey procedures, sampling design and technique, and inclusion/exclusion criteria are delineated elsewhere [23]. We selected all nationally representative datasets that included the key variables pertaining to the analysis. For countries with more than one GSHS dataset, we used the most recent one available. The analytical sample consists of 207,711 participants aged 11 to 17 years from 71 countries. The GHS data is publicly available online.

### Outcome variable

We treated adolescent CSA as an outcome variable. CSA was defined as missing 10% (or 18 days) of a school year [4, 5]. The outcome variable was measured with a single self-reported item or question “During the past 30 days, on how many days did you miss classes or school without permission?”, including response options: 0 days, 1 or 2 days, 3 to 5 days, 6 to 9 days, 10 or more days. The response was dichotomised (1 = ‘yes’ if the participants reported at least three days or more missed school for any reasons during the past 30 days or 0 = ‘no’ otherwise).

### Explanatory variables

The following explanatory variables were included in the present study. Violence and unintentional injury were assessed with the questions in the measure (how often students have been physically attacked, how often they have participated in a physical fight, frequency of serious injuries, and frequency of bullying). Physical violence by peer’s was assessed with the questions: “During the past 12 months, how many times were you physically attacked” and “During the past 12 months, how many times were you in a physical fight?”. Student responses for being physically attacked and fighting were recoded as ‘yes’ (reported being attacked or fighting one or more times) or ‘no’ otherwise. The status of student’s serious injuries was defined as ‘yes’ if they reported being seriously injured one or more times according to the question “During the past 12 months, how many times were you seriously injured?” or ‘no’ otherwise. Participants’ bullying victimisation was defined as dichotomised (1 = ’yes’ if the participant reported bullying experiences on one or more days, or 0 = ’no’ otherwise). Two psychological factors included in this study were anxiety and loneliness. Participant’s level of anxiety and loneliness were measured using the following questions: ‘During the past 12 months, how often have you been so worried about something that you could not sleep at night?’ and ‘During the past 12 months, how often have you felt lonely?’. These responses were coded as never, rarely or sometimes, most of the time, or always.

Peer support was assessed using a proxy variable based on the question ‘During the past 30 days, how often were most of the students in your school kind and helpful?’ to which students could respond ‘never’, ‘rarely’, ‘sometimes’, ‘most of the time’ or ‘always’. Responses were recoded as 0 = never, 1 = rarely or sometimes, or 2 = most of the times and always. The number of close friends were recorded as: 0 = none, 1 = 1–2 friends, or 2 = ≥ 3 friends based on the survey question ‘How many close friends do you have?’. Parental regulation and monitoring were assessed as the role of parental support using three variables such as parents checking homework (i.e., ‘During the past 30 days, how often did your parents or guardians check to see if your homework was done?’), parents understanding the problem (i.e., ‘During the past 30 days, how often did your parents or guardians understand your problems and worries?’), and parental monitoring (i.e., ‘During the past 30 days, how often did your parents or guardians really know what you were doing with your free time?’). Responses were recorded as never, rarely or sometimes, most of the time, or always.

Respondent’s food insecurity was measured according to the following survey question: ‘During the past 30 days, how often did you go hungry because there was not enough food in your home?’. Responses were recoded ‘most of the time or always’ as ‘poor food security (Q_1_)’, ‘rarely or sometimes’ as ‘average of food security (Q_2_)’, and ‘never’ as ‘high food security (Q_3_)’. In addition, participants were asked about time spent engaged in sitting activities and watching television as well as their weight and height. Students were asked the following question: ‘How much time do you spend during a typical or usual day sitting and watching television, playing computer games, talking with friends, or doing other sitting activities. Student’s everyday sitting activities were categorised as follows: none, <1 hour, 1–2 hours, 3–4 hours, and 5 hours or more as added the survey question. Height and weight data were self-reported rather than measured. The body mass index (BMI) was calculated for each participant as weight divided by height squared (kg/m^2^) and then computed age- and sex specific z-score relative to the BMI-for-age using the 2007 World Health Organization standards [24]. Study participants were classified as normal BMIz if ’-2·00 standard deviation (SD) < BMIz <1·00 SD’, overweight if ’1·00 SD < BMIz ≤ 2·00 SD’, obesity if ’BMIz > 2·00 SD’, and overweight or obesity if ’BMIz ≥ 1·00 SD.

Two demographic factors were included as independent variables. Age was grouped as follows: 11–12 years, 13 years, 14 years, 15 years, 16 years, and 17 years. The sex of the participants was coded as male and female.

Based on the literature review we selected the above explanatory variables.

### Statistical analysis

Due to the complex nature of the data, a composite samples’ option was applied in the analytical exploration, accounting for country-specific primary sampling unit, stratum, and sample weight. All analyses were weighted using sampling unit (PSU) which is derived from the probability of a school being selected, a classroom being selected, school and student level non-response and gender. Therefore, the samples were representative in respect to the study population. This included using strata and primary sampling units at the country-specific data. Weighted estimates of prevalence were expressed with corresponding 95% confidence intervals (CIs) for the national and regional perspectives. Univariable binary logistic regression was conducted to estimate the crude effect followed by multivariable logistic regression to estimate the adjusted effect along with 95% confidence interval were employed to determine the associated factors of CSA. The variables found significant at (p<0.05) in the univariable models were taken for multivariable analysis. A p-value of less than 0.05 was considered as statistically significant. Multicollinearity among covariates was checked using variance inflation factor (VIF). All analyses were performed using the statistical software Stata/SE 13 (StataCorp, College Station, Texas, USA).

### Patient and public involvement

This study is based on secondary data. There was no patient or public involvement.

## Results

### Participant’s characteristics

The participants’ mean age 13·43 (SD 2·16), and (47·77%) of them were males. Almost half of the adolescents reported to have food insecurity at home most often or sometimes (46·25%), ever suffering from bullying (32·18%), high levels of anxiety (9·61%), loneliness (12·34%), being physically attacked (33·89%), poor peer support (14·48%), and not having close friends (7·93%). The prevalence of suicidal ideation, suicidal plan and suicidal attempts among adolescents was 17·70%, 16·18%, and 16·08%, respectively. Approximately, 39·47% of adolescent’s parent checked homework and 38·81% parents understood adolescent’s problem most often, and 44·86% of parents monitored their adolescent’s activities at home and outside.

### Prevalence of adolescents’ CSA

**Fig 1:**
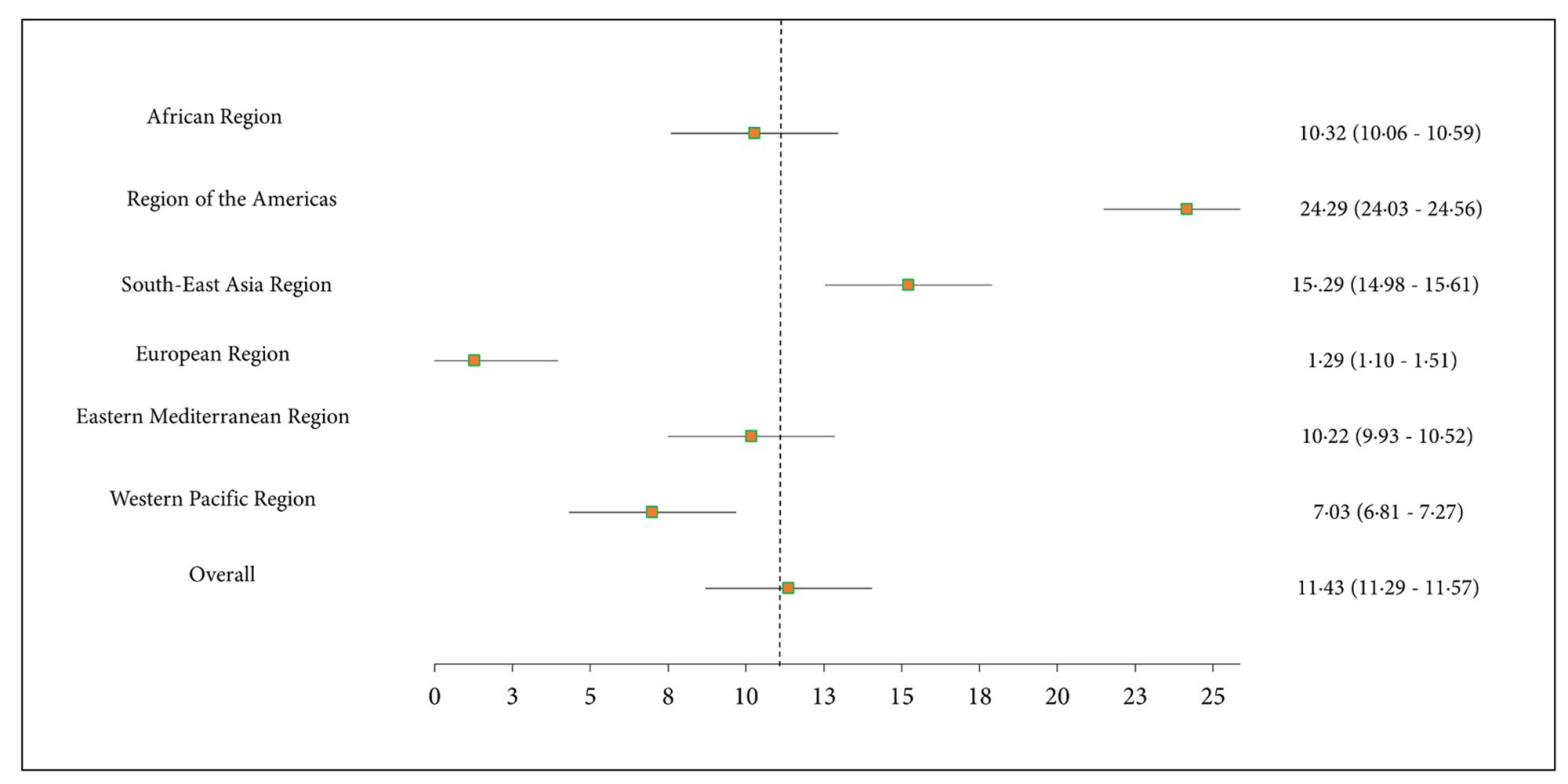
Pooled prevalence of chronic school absenteeism.

**Fig 2:**
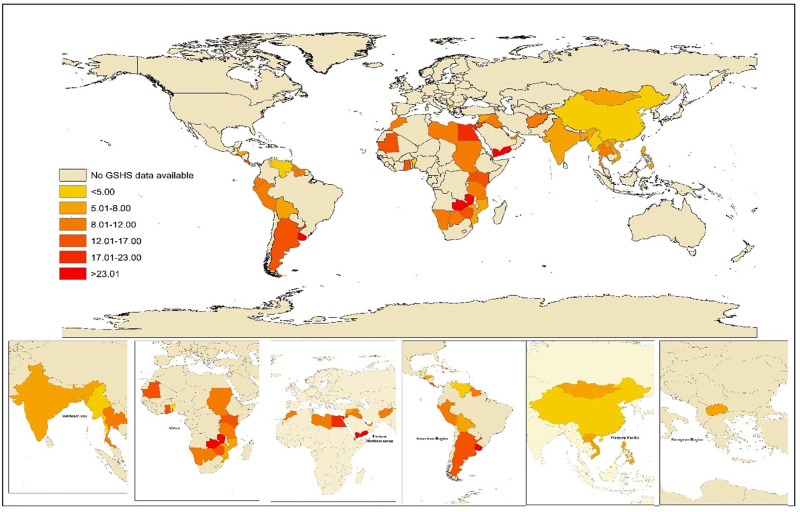
Global pooled prevalence of chronic school absenteeism in the 1– months preceding survey completion among adolescents aged 11–17 years for 71 low–middle–and high–income countries, 2003–2015. A change in colour from yellow to red exhibits a greater magnitude of chronically absent adolescents at national, regional and global levels.

The overall pooled prevalence of CSA was 11·43% (95% CI: 11·29–11·57) among the included school-aged adolescents (Fig 1). The sex-specific geographic distribution of CSA prevalence is illustrated in Fig 2 and S1 and S2 Figs in S1 File. The distribution of CSA prevalence by participants’ characteristics are also presented in Table 1.

**Table 1:** Participant’s background characteristics.

Characteristics	Number of participants—n (%)	Prevalence of CSA—% (95% CI)
**Age in years**		
11–12 years	17066 (8.31)	9.01 (8.59–9.45)
13 years	38653 (18.81)	9.31 (9.03–9.60)
14 years	51501 (25.07)	10.79 (10.52–11.06)
15 years	48698 (23.70)	12.15 (11.86–12.44)
16 years	36247 (17.64)	14.86 (14.49–15.23)
17 years	13279 (6.46)	10.26 (9.75–10.78)
**Sex of the student**		
Male	97955 (47.77)	12.8 (12.59–13.01)
Female	107118 (52.23)	9.98 (9.81–10.17)
**Food insecurity**		
None	104919 (53.74)	10.13 (9.95–10.32)
Sometimes or rarely	76260 (39.06)	11.95 (11.72–12.18)
Most of time or always	14043 (7.19)	18.31 (17.68–18.96)
**Bullied**		
No	122007 (67.82)	9.32 (9.16–9.49)
Yes	57891 (32.18)	15.71 (15.41–16.01)
**Loneliness**		
Never	70904 (35.59)	9.54 (9.33–9.76)
Sometimes or rarely	103721 (52.07)	10.76 (10.58–10.95)
Most of time or always	24589 (12.34)	17.06 (16.60–17.54)
**Anxiety**		
Never	68401 (35.81)	9.81 (9.59–10.03)
Sometimes or rarely	104235 (54.58)	10.67 (10.48–10.86)
Most of time or always	18355 (9.61)	18.81 (18.25–19.38)
**Physically attacked**		
No	110343 (66.11)	8.54 (8.38–8.71)
Yes	56563 (33.89)	18.31 (17.99–18.63)
**Physical fighting**		
No	129252 (64.47)	7.67 (7.52–7.81)
Yes	71247 (35.53)	18.28 (18.00–18.57)
**Seriously injured**		
No	96714 (59.16)	8.89 (8.71–9.07)
Yes	66768 (40.84)	15.53 (15.26–15.81)
**Peer supports**		
Never	29588 (14.48)	14.09 (13.70–14.49)
Sometimes or rarely	93092 (45.57)	11.47 (11.27–11.68)
Most of time or always	81616 (39.95)	9.83 (9.62–10.03)
**Number of close friends**		
None	15463 (7.93)	13.12 (12.59–13.66)
1–2 friends	61967 (31.77)	10.88 (10.63–11.12)
≥ 3 friends	117646 (60.31)	10.88 (10.71–11.06)
**Parents check homework**		
Never	49539 (24.35)	12.41 (12.12–12.7)
Sometimes or rarely	73609 (36.18)	12.71 (12.47–12.95)
Most of time or always	80313 (39.47)	8.66 (8.47–8.86)
**Parent understand problem**		
Never	48141 (23.72)	13.30 (13.00–13.61)
Sometimes or rarely	76039 (37.47)	12.01 (11.78–12.24)
Most of time or always	78773 (38.81)	8.70 (8.50–8.90)
**Parent monitoring**		
Never	39477 (19.54)	12.82 (12.50–13.16)
Sometimes or rarely	71902 (35.60)	12.41 (12.17–12.65)
Most of time or always	90611 (44.86)	9.07 (8.88–9.26)
**Sitting activities per day**		
<1 hour	64793 (32.82)	9.57 (9.34–9.80)
1–2 hours	62114 (31.46)	9.27 (9.04–9.50)
3–4 hours	37331 (18.91)	12.04 (11.71–12.37)
>4 hours	33195 (16.81)	18.19 (17.78–18.61)
**Adolescent obesity status**		
Normal weight	175278 (84.50)	11.20 (11.05–11.35)
Overweight	22492 (10.84)	12.32 (11.90–12.76)
Obesity	9662 (4.66)	13.50 (12.83–14.19)
**Total observation–N**	**207711 (100)**	**11.43 (11.29–11.57)**

### Factors associated with adolescents’ CSA

In univariable regression analyses, adolescents with older age food insecurity, victimisation, loneliness, anxiety, physical attack, physical fighting, serious injury, less peer support, fewer close friends, parents checking homework less often, parents understanding problems less often, parents monitoring less often, overweight or obesity, and sitting for more hours per day were significantly associated with higher odds of chronic school absenteeism (all ORs>1, all p<0.05), whereas female adolescents were associated with lower odds than males (OR = 0.76, 95% CI: 0.74–0.78). The results from multivariable regression analyses were mostly consistent with univariable analyses, except that victimisation was no longer a significant factor (OR = 1.02, 95% CI: 0.98–1.07) and the odds of chronic school absenteeism were relatively lower in adolescents that reported sometimes or rarely food insecurity (vs no food insecurity: OR = 0.90, 95% CI: 0.86–0.94), no peer support (vs peers were always supportive: OR = 0.93, 95% CI: 0.89–0.97), fewer close friends (1–2 friends vs 3 or more friends: OR = 0.88, 95% CI: 0.84–0.93), or sitting activities for 1–2 hours per day (vs sitting activities for less than 1 hour per day: OR = 0.91, 95% CI: 0.86–0.96) (Table 2).

**Table 2:** Association of adolescent’s chronic absenteeism at school and associated factors for the global perspective.

Characteristics	Likelihood of adolescent’s chronic absenteeism at school			
	Crude model		Adjusted model	
	OR (95% CI)	P-value	OR (95% CI)	P-value
**Age in years (ref = 11–12 years)**				
13 years	1·04 (0·97–1·10)	0·252	1·25 (1·13–1·39)	<0·0001
14 years	1·22 (1·15–1·30)	<0·0001	1·56 (1·41–1·73)	<0·0001
15 years	1·40 (1·32–1·48)	<0·0001	1·77 (1·60–1·95)	<0·0001
16 years	1·76 (1·66–1·87)	<0·0001	2·25 (2·03–2·49)	<0·0001
17 years	1·15 (1·07–1·25)	<0·0001	1·74 (1·54–1·96)	<0·0001
**Female (ref = Male)**	0·76 (0·74–0·78)	<0·0001	0·96 (0·92–1·00)	0·045
**Food insecurity (ref = None)**				
Sometimes or rarely	1·20 (1·17–1·24)	<0·0001	0·90 (0·86–0·94)	<0·0001
Most of time or always	1·99 (1·90–2·08)	<0·0001	1·22 (1·13–1·32)	<0·0001
**Peer victimisation (ref = no)**	1·81 (1·76–1·87)	<0·0001	1·02 (0·98–1·07)	0·321
**Loneliness (ref = never)**				
Sometimes or rarely	1·14 (1·11–1·18)	<0·0001	0·96 (0·92–1·01)	0·145
Most of time or always	1·95 (1·87–2·03)	<0·0001	1·12 (1·05–1·20)	0·001
**Anxiety (ref = never)**				
Sometimes or rarely	1·10 (1·06–1·13)	<0·0001	1·07 (1·02–1·12)	0·009
Most of time or always	2·13 (2·04–2·23)	<0·0001	1·45 (1·35–1·55)	<0·0001
**Physically attacked (ref = no)**	2·40 (2·33–2·47)	<0·0001	1·66 (1·58–1·73)	<0·0001
**Physically fighting (ref = no)**	2·69 (2·62–2·77)	<0·0001	2·24 (2·15–2·35)	<0·0001
**Seriously injured (ref = no)**	1·89 (1·83–1·94)	<0·0001	1·06 (1·02–1·11)	0·005
**Peer were supportive (ref = most of time or always)**				
Never	1·50 (1·45–1·57)	<0·0001	0·93 (0·89–0·97)	0·002
Sometimes or rarely	1·19 (1·15–1·23)	<0·0001	1·20 (1·13–1·27)	<0·0001
**Number of close friends (ref = ≥ 3)**				
None	1·24 (1·18–1·30)	<0·0001	0·93 (0·86–1·01)	0·075
1–2 friends	1·00 (0·97–1·03)	<0·0001	0·88 (0·84–0·93)	<0·0001
**Parents check homework (ref = most of time or always)**				
Never	1·49 (1·44–1·55)	<0·0001	1·22 (1·15–1·29)	<0·0001
Sometimes or rarely	1·54 (1·49–1·59)	<0·0001	1·35 (1·28–1·42)	<0·0001
**Parent understand problem (ref = most of time or always)**				
Never	1·61 (1·55–1·67)	<0·0001	1·43 (1·35–1·51)	<0·0001
Sometimes or rarely	1·43 (1·39–1·48)	<0·0001	1·14 (1·08–1·20)	<0·0001
**Parent monitoring (ref = most of time or always)**				
Never	1·47 (1·42–1·53)	<0·0001	0·94 (0·89–1·00)	0·065
Sometimes or rarely	1·42 (1·38–1·47)	<0·0001	1·06 (1·01–1·11)	0·021
**Adolescent obesity status (ref = normal weight)**				
Overweight	1·11 (1·07–1·16)	<0·0001	1·11 (1·04–1·18)	0·001
Obesity	1·24 (1·16–1·31)	<0·0001	1·17 (1·07–1·28)	<0·0001
**Sitting activities per day (ref = <1 hour)**				
1–2 hours	0·97 (0·93–1·00)	0·068	0·91 (0·86–0·96)	<0·0001
3–4 hours	1·29 (1·24–1·35)	<0·0001	1·03 (0·97–1·10)	0·275
>4 hours	2·10 (2·02–2·18)	<0·0001	1·50 (1·42–1·59)	<0·0001
**Constant**	-	-	0·03 (0·02–0·03)	<0·0001
**Linktest (hat OR)**			2·61 (2·29–2·98)	<0·0001
**Hosmer-Lemeshow χ**^**2**^ **statistic**			86·61	<0·0001
**Area under ROC curve**			0·83	
**Mean VIF (Maximum)**			1·87 (2·81)	

OR, Odds Ratio; CI, Confidence Interval; VIF, Variance Inflation Factor.

### Factors associated with adolescents’ CSA by region

Table 3 describes the factors associated with adolescent CSA by 6 WHO regions. Adolescents aged more than 12 years were associated with higher likelihood of CSA (all ORs>1, all p<0.05), and female adolescents were significantly associated with 4% lower odds of CSA compared to male counterpart (OR = 0.96, 95% CI: 0.92–0.99). In the context of WHO regions, older age was generally associated with increased odds of CSA among adolescents in the Americas (OR = 4.20, 95% CI: 3.56–4.95, age 16, y) and Western Pacific (OR = 2.12, 95% CI: 1.60–2.81, in age 17, y), but was significantly related to lower odds in Africa (OR = 0.62, 95% CI: 0.48–0.80, in age 16, y) and Eastern Mediterranean (OR = 0.60, 95% CI: 0.47–0.78 in age 13, y).

**Table 3:** Association (adjusted regression) of adolescent’s chronic absenteeism at school and associated factors by region.

Characteristics	African		Americas		South–East Asia		European		Eastern Mediterranean		Western Pacific	
	OR (95% CI)	P–value	OR (95% CI)	P–value	OR (95% CI)	P–value	OR (95% CI)	P–value	OR (95% CI)	P–value	OR (95% CI)	P–value
Age in years (ref = 11–12 years)												
13 years	0·81 (0·62–1·05)	0·116	1·77 (1.50–2.10)	<0.0001	1.00 (0.63–1.59)	0.990	1.35 (0.17–10.88)	0.778	0.60 (0.47–0.78)	<0.0001	1.30 (1.00–1.69)	0.053
14 years	0.73 (0.56–0.95)	0.017	2.37 (2.01–2.79)	<0.0001	1.00 (0.64–1.58)	0.994	1.34 (0.17–10.71)	0.785	0.70 (0.55–0.89)	0.004	1.44 (1.12–1.86)	0.005
15 years	0.58 (0.45–0.75)	<0.0001	2.88 (2.44–3.39)	<0.0001	0.87 (0.54–1.40)	0.554	2.19 (0.28–16.89)	0.453	0.92 (0.72–1.17)	0.493	1.50 (1.16–1.93)	0.002
16 years	0.62 (0.48–0.80)	<0.0001	4.20 (3.56–4.95)	<0.0001	1.17 (0.70–1.96)	0.550	4.89 (0.64–37.19)	0.125	1.20 (0.94–1.54)	0.143	1.69 (1.30–2.18)	<0.0001
17 years	1.05 (0.82–1.33)	0.713	3.67 (2.78–4.84)	<0.0001	1.24 (0.73–2.09)	0.431	4.27 (0.39–46.17)	0.232	1.18 (0.88–1.59)	0.258	2.12 (1.60–2.81)	<0.0001
Female (ref = Male)	0.87 (0.77–0.99)	0.031	1.04 (0.98–1.10)	0.189	0.66 (0.51–0.83)	0.001	1.04 (0.64–1.70)	0.882	0.83 (0.72–0.94)	0.004	0.74 (0.67–0.83)	<0.0001
Food insecurity (ref = None)												
Sometimes or rarely	1.09 (0.95–1.25)	0.242	0.93 (0.87–0.98)	0.012	0.96 (0.75–1.22)	0.726	0.97 (0.54–1.76)	0.925	0.95 (0.83–1.09)	0.498	1.19 (1.05–1.34)	0.005
Most of time or always	1.47 (1.22–1.78)	<0.0001	1.10 (0.96–1.25)	0.167	2.12 (1.44–3.10)	<0.0001	2.23 (0.47–10.60)	0.314	1.29 (1.06–1.57)	0.011	2.10 (1.75–2.50)	<0.0001
Peer victimisation (ref = no)	1.32 (1.15–1.51)	<0.0001	0.93 (0.87–0.99)	0.018	1.34 (1.03–1.75)	0.030	1.24 (0.65–2.39)	0.510	1.25 (1.09–1.43)	0.001	1.53 (1.35–1.73)	<0.0001
Loneliness (ref = never)												
Sometimes or rarely	0.88 (0.76–1.02)	0.102	1.01 (0.95–1.08)	0.775	0.84 (0.65–1.10)	0.216	1.13 (0.66–1.94)	0.646	1.05 (0.91–1.22)	0.471	0.92 (0.80–1.05)	0.225
Most of time or always	1.00 (0.82–1.21)	0.972	1.13 (1.03–1.25)	0.012	1.07 (0.72–1.58)	0.741	0.63 (0.21–1.89)	0.407	1.30 (1.08–1.56)	0.005	1.12 (0.93–1.34)	0.235
Anxiety (ref = never)												
Sometimes or rarely	1.18 (1.01–1.37)	0.031	1.01 (0.95–1.07)	0.858	0.95 (0.73–1.25)	0.733	0.91 (0.53–1.54)	0.713	0.96 (0.83–1.11)	0.592	1.08 (0.95–1.23)	0.254
Most of time or always	1.82 (1.51–2.20)	<0.0001	1.34 (1.21–1.49)	<0.0001	1.79 (1.19–2.68)	0.005	2.02 (0.82–4.92)	0.124	1.28 (1.06–1.54)	0.010	1.53 (1.28–1.84)	<0.0001
Physically attacked (ref = no)	1.33 (1.16–1.52)	<0.0001	1.77 (1.67–1.89)	<0.0001	1.34 (1.03–1.75)	0.029	1.27 (0.73–2.21)	0.398	1.16 (1.01–1.33)	0.030	1.41 (1.25–1.59)	<0.0001
Physically fighting (ref = no)	1.64 (1.43–1.87)	<0.0001	2.38 (2.24–2.53)	<0.0001	1.31 (1.00–1.72)	0.052	2.08 (1.29–3.35)	0.003	1.42 (1.24–1.62)	<0.0001	1.60 (1.42–1.81)	<0.0001
Seriously injured (ref = no)	1.45 (1.26–1.67)	<0.0001	0.99 (0.93–1.05)	0.707	1.75 (1.36–2.26)	<0.0001	1.54 (0.97–2.47)	0.070	1.33 (1.17–1.51)	<0.0001	1.59 (1.40–1.79)	<0.0001
Peer were supportive (ref = most of time or always)												
Never	1.00 (0.83–1.20)	<0.0001	1.37 (1.25–1.49)	<0.0001	1.91 (1.33–2.73)	<0.0001	1.63 (0.81–3.28)	0.169	1.36 (1.13–1.63)	0.001	0.92 (0.76–1.10)	0.350
Sometimes or rarely	0.92 (0.79–1.06)	0.241	1.04 (0.98–1.11)	0.193	1.36 (1.04–1.78)	0.026	1.15 (0.69–1.89)	0.596	1.17 (1.02–1.33)	0.027	1.03 (0.92–1.17)	0.587
Number of close friends (ref = ≥ 3)												
None	0.97 (0.79–1.20)	0.792	0.91 (0.81–1.02)	0.097	1.11 (0.72–1.70)	0.650	0.73 (0.16–3.30)	0.683	1.18 (0.94–1.48)	0.151	1.14 (0.92–1.42)	0.220
1–2 friends	0.87 (0.76–0.99)	0.042	0.85 (0.80–0.91)	<0.0001	1.09 (0.84–1.42)	0.504	1.10 (0.65–1.84)	0.727	1.06 (0.92–1.21)	0.412	1.19 (1.05–1.35)	0.006
Parents check homework (ref = most of time or always)												
Never	1.50 (1.27–1.78)	<0.0001	1.13 (1.05–1.22)	0.002	1.06 (0.77–1.47)	0.717	1.02 (0.57–1.81)	0.951	1.01 (0.86–1.18)	0.942	1.25 (1.07–1.46)	0.005
Sometimes or rarely	1.25 (1.07–1.45)	0.005	1.36 (1.27–1.46)	<0.0001	0.99 (0.74–1.33)	0.971	0.94 (0.55–1.61)	0.818	1.18 (1.02–1.36)	0.027	1.07 (0.94–1.22)	0.327
Parent understand problem (ref = most of time or always)												
Never	0.94 (0.78–1.13)	0.524	1.78 (1.64–1.92)	<0.0001	0.93 (0.66–1.30)	0.663	0.90 (0.40–2.03)	0.796	0.92 (0.77–1.09)	0.330	0.73 (0.62–0.87)	<0.0001
Sometimes or rarely	1.11 (0.96–1.29)	0.168	1.25 (1.16–1.34)	<0.0001	0.78 (0.59–1.05)	0.097	1.22 (0.74–1.99)	0.436	1.00 (0.86–1.16)	0.984	0.95 (0.83–1.09)	0.494
Parent monitoring (ref = most of time or always)												
Never	1.26 (1.05–1.52)	0.011	0.94 (0.86–1.02)	0.113	1.12 (0.78–1.62)	0.536	2.53 (1.13–5.66)	0.024	1.42 (1.19–1.69)	<0.0001	1.37 (1.16–1.62)	<0.0001
Sometimes or rarely	1.24 (1.07–1.45)	0.005	1.03 (0.97–1.10)	0.294	1.24 (0.94–1.64)	0.133	2.41 (1.47–3.93)	<0.0001	1.64 (1.41–1.90)	<0.0001	1.27 (1.12–1.45)	<0.0001
Adolescent obesity status (ref = normal weight)												
Overweight	0.97 (0.77–1.22)	0.784	0.98 (0.90–1.06)	0.556	1.36 (0.91–2.05)	0.136	1.04 (0.49–2.20)	0.919	1.09 (0.92–1.29)	0.343	1.34 (1.14–1.58)	<0.0001
Obesity	0.87 (0.63–1.21)	0.418	1.04 (0.92–1.17)	0.549	0.94 (0.45–1.98)	0.878	0.85 (0.10–7.09)	0.884	1.11 (0.89–1.37)	0.348	1.63 (1.34–1.98)	<0.0001
Sitting activities per day (ref = <1 hour)												
1–2 hours	1.25 (1.06–1.46)	0.008	0.81 (0.76–0.87)	<0.0001	0.74 (0.53–1.04)	0.081	0.75 (0.34–1.64)	0.468	0.93 (0.77–1.12)	0.425	1.22 (1.06–1.41)	0.005
3–4 hours	1.63 (1.35–1.96)	<0.0001	0.79 (0.73–0.86)	<0.0001	1.33 (0.95–1.86)	0.102	1.13 (0.54–2.34)	0.746	1.36 (1.13–1.64)	0.001	1.65 (1.41–1.92)	<0.0001
>4 hours	2.48 (2.10–2.93)	<0.0001	1.11 (1.03–1.20)	0.007	1.46 (1.05–2.03)	0.026	1.39 (0.68–2.87)	0.370	2.91 (2.45–3.44)	<0.0001	2.15 (1.85–2.50)	<0.0001

OR, Odds Ratio; CI, Confidence Interval.

Adolescents who reported food insecurity often or always had increased odds of being chronically absent from school across regions (Africa: OR = 1.47, 95% CI: 1.22–1.78), (South–East Asia: OR = 2.12, 95% CI: 1.44–3.10), (Eastern Mediterranean: OR = 1.29, 95% CI: 1.06–1.57), (Western Pacific: OR = 2.10, 95% CI: 1.75–2.50). High levels of sedentary behaviour was linked to an increased risk of CSA in African regions (OR = 2.48, 95% CI: 2.10–2.93, >4 hours; OR = 1.63, 95% CI: 1.35–1.96, 3–4 hours) and Eastern Mediterranean (OR = 2.91,95% CI: 2.45–3.44, >4 hours; OR = 1.36, 95% CI: 1.13–1.64, 3–4 hours) and Western Pacific (OR = 2.15, 95% CI: 1.85–2.50, >4 hours; OR = 1.65, 95% CI: 1.41–1.92, 3–4 hours), South–East Asia (OR = 1.46, 95% CI: 1.05–2.03, >4 hours).

In most regions, adolescent victimisation by peer groups was associated with an increased likelihood of chronic absence from school (African: OR = 1.32, 95% CI: 1.15–1.51); (South–East Asia: OR = 1.34, 95% CI: 1.03–1.75); (Western Pacific: OR = 1.53 95% CI: 1.35–1.73) and (Eastern Mediterranean: OR = 1.25, 95% CI: 1.09–1.43). Adolescents who were physically attacked were more likely to be chronically inattentive at school in most regions, with the exception of Europe. African adolescents (OR = 1.33, 95% CI: 1.16–1.52), American (OR = 1.77, 95% CI: 1.67–1.89, p<0.0001), South–East Asian (OR = 1.34, 95% CI: 1.03–1.75, p = 0.029), Eastern Mediterranean (OR = 1.16, 95% CI: 1.01–1.33) and Western Pacific (OR = 1.41, 95% CI: 1.25–1.59) were more profound in missing school chronically. In most regions, being seriously physically injured was linked to an increased odds of chronic absenteeism among adolescents, with the exception of the American and European regions. Adolescents who engaged in physical fighting had high odds of chronic absenteeism in all regions except South–East Asia.

Loneliness was not found to be a significant risk factor for CSA, except in the Americas (OR = 1.13, 95% CI: 1.03–1.25) and Eastern Mediterranean (OR = 1.30, 95% CI: 1.08–1.56), where those reporting always lonely were linked to significantly higher odds than those reporting never lonely. In all regions except Europe, adolescents with anxiety often/always were associated with increased odds of chronic school absenteeism compared to adolescents who never experienced anxiety (African: OR = 1.82, 95% CI: 1.51–2.20); (American: OR = 1.34, 95% CI: 1.21–1.49); (South–East Asia: OR: 1.79, 95% CI: 1.19–2.68); (Eastern Mediterranean: OR: 1.28, 95% CI: 1.06–1.54) and (Western Pacific: OR = 1.53, 95% CI: 1.28–1.84).

Adolescents without supportive parents were more likely to be chronically absent from school than those who had supportive parents in most regions (American: OR = 1.37, 95% CI: 1.25–1.49); (South–East Asia: OR = 1.91, 95% CI: 1.33–2.73); (Eastern Mediterranean: OR = 1.36, 95% CI: 1.13–1.63), with the exception of Europe and the Western Pacific. Furthermore, peer support for adolescents in Southeast Asia and the Eastern Mediterranean minimised the chance of CSA at 36% to 17%, respectively. In Africa (OR = 1.50, 95% CI: 1.27–1.78), America (OR = 1.13, 95% CI: 1.05–1.22), and Western Pacific (OR = 1.25, 95% CI: 1.07–1.46), parents who had never checked homework were associated with a considerably higher likelihood of CSA, but not in South–East Asia or Europe. Adolescents with parents who did not understand the former’s problem had higher odds of CSA in America (OR = 1.78, 95% CI: 1.64–1.92) than adolescents with parents who understood their problems (Table 3).

## Discussion

Males were found to report higher CSA than females which is consistent with a previous study [25] but dissimilar to other studies where females were reported to have more prevalence of CSA [26, 27]. Across regions with the exception of America and Europe, male adolescents had higher extent of CSA relative to female adolescents. This regional variation could be a reflection of various efforts to keep girls in school as a pathway to female empowerment in different parts of the world. Nonetheless, in some contexts, girls may find it difficult to attend school due to a lack of menstrual education, limited access to sanitary supplies, and an unpleasant school environment combined with cultural constraints such as early marriage and family unwillingness to invest in girl–child education result in a higher female CSA in comparison with males [28–32].

We found higher CSA among food insecure adolescents across regions, except for Americas and Europe. This finding is supported by previous studies of school absenteeism [33–35]. Persistent food insecurity translates to hunger during school periods which challenges the food security, social inclusion and anti- poverty interventions globally. However, the Americas and Europe, have made noteworthy progress in terms of social inclusion and the eradication of extreme poverty which consequently reduce food insecurity and lead to less chronic absences of adolescents. On the other hand, we found high CSA in Africa and Asian regions often reported to have moderate to severe food insecurity problems [36]. We argue that countries in the African, Southeast Asian, and Western Pacific regions must devote substantial resources to combating persistent food insecurity in order to reduce CSA prevalence. Hence, national policies and initiatives should accelerate ways to boost family income generating ability and social-economic status in order to address household food insecurity. In addition, school feeding interventions can be initiated to tackle CSA. School feeding has been positively associated with decreasing adolescents school absenteeism [37, 38].

Higher level of sedentary behaviours (≥ 4 hours per day) increased the odds of CSA from school. However, our regional analysis depicts both obesity and sedentary behaviours (≥1 hour per day) were significantly associated with CSA in only Western Pacific. Sedentary behaviours (≥1 hour per day) significantly increased odds of CSA among adolescents in all the regions apart from Europe. Our findings corroborate an earlier study that reported similar findings in relation to sitting activities per day and CSA among adolescents [39]. Comparably, the findings on adolescent obesity and CSA are consistent with previous research on the subject [40, 41]. The ill-health conditions associated with obesity may be a possible explanation for the high risk of CSA among obese adolescents [41].

We found physical fighting, peer conflict, and serious injury among adolescents strongly linked to the proliferation of CSA, which is consistent with previous research [6, 42]. Physical attacks and physical fighting influenced absence from school except Europe and South-East Asia, on other hand, being seriously injured augmented CSA in all regions excluding America and Europe. The reason might be students who have been physically attacked or who have repeatedly participated in physical fights may be unable to attend school; additionally, they may feel uncomfortable and unsafe in a school environment, or they may be suspended from school. The reasons for the regional differences, however, are unknown and require further cohort investigation.

School is a vital social environment, but it can also be a terrifying place for those who suffer from loneliness, anxiety, or have strained social relationships. The unfavourable experiences may be linked to a hatred for school, which can have a detrimental impact on a student’s motivation, academic progress, overall well-being and frequent missing of school. We have found adolescents who have anxiety were more likely to frequently miss classes which is consistent with previous studies [43–47]. These findings suggest some children may enter school with individual risk factors for absenteeism, such as anxiety, or negative parental attitudes regarding school accomplishment and attendance. Learning difficulties throughout time, as well as an unpleasant or unsatisfactory school or classroom environment, may further increase the CSA. Therefore, merely treating anxiety issues may not be enough for school refusers; early recognition of behavioural disorders, familial issues, and participation in prosocial activities may all be beneficial and minimise the likelihood of adolescents’ CSA. Interestingly, loneliness was more pronounced in the Americas and Eastern Mediterranean regions, possibly due to the stratums of social inequality, increased substitution of the virtual world (e.g. use of computers and social media) with reality [48, 49].

We found parental involvement with adolescents in LMICs statistically important with regard to CSA. Adolescents whose parents never or seldom reviewed their homework, understood their problems, monitored academic performance or observed their adolescents’ movements had substantially high odds of CSA. This finding affirms previous studies findings on CSA [50, 51]. Nonetheless, we observed regional variations in America and Southeast Asia where the CSA was considerably greater among students whose parents were less close to their children. In Africa and the Americas, adolescents whose parents inspected their homework less often were more likely to encounter CSA, but not in the other regions. These results highlight the importance of region-specific initiatives aimed at boosting parental participation in their children’s education.

We assert that lack of peer and parental support is linked to an increased risk of CSA, our study shows adolescents without peer support and close friends (1 or 2) increased the odds of CSA which is corroborated by a previous study [52]. The reasonable explanation would be that, adolescents with positive peer support or more friends may be encouraged to stay out of school. Furthermore, all other factors being equivalent, an adolescent who has never received peer support is unlikely to determine how peer support influences CSA, and therefore will be less likely to relate CSA to peer support. This is exemplified in our findings that compared adolescents who mostly had peer support to those who sometimes or rarely received peer support were less likely to be chronically absent from school. Peer groups may aid in the reduction of anti-social behaviour, which is important in encouraging school attendance [53].

This study revealed a high level of CSA and identified several risks and protective factors among school-based adolescents. We present insight into the development of effective national and global policies to prevent adolescent CSA. School authorities can play a key role in preventing violence and unintentional injury (e.g., being physically attacked, participating in physical fighting, being seriously injured, and being bullied victimisation by peers) through positive youth development programs [54] that that enable the development of intentional self-regulation and multiple positive assets. Parents should be responsible for building a protective, caring and loving home environment to improve social responsibility, positive awareness and behaviours, and enhance social-interpersonal relationships. Schools and communities need to be supported to build a safe and child-friendly environment outside the adolescent’s home [55].

### Strengths and limitations

The study’s strengths include a high sample size of adolescents from 71 LMICs across six WHO regions, with the majority of these samples being nationally representative. The GSHS employed consistent techniques across surveys, including sample size (e.g., school-based), data collection procedures, and question wording, allowing for accurate analyses of cross-national or regional disparities [56]. The results given in this publication were derived by weighted analyses in which the GSHS weighting factored in the population’s gender and age distributions. Weighting was utilised to guarantee that the results were generalizable to the whole target population, not simply those who responded to the survey. As a result, any skewness in the observed data by sex (or age) is unlikely to have an effect on the outcomes of the weighted analysis.

Nonetheless, the following limitations should be considered when interpreting findings. There is scant evidence of the GSHS measures’ reliability and validity across cultural contexts. Due to the cross-sectional nature of the data, risk and protective factors were collected concurrently, thus causality between risk and protective factors cannot be inferred. In addition, self -reporting questionnaires are subject to social desirability and recall bias which can increase or decrease the strength the observed association between the outcome and explanatory variables. Besides, the information about the adolescents who were absent on the day of data collection was not available that may also affect the precision of generalizability of the findings. Some respondents may have struggled to comprehend the questionnaire (e.g., due to weak reading skills), and similarly, parental authorization for offspring to participate is contingent upon parental literacy skills [56]. Countries were allowed to use translated versions of the GSHS; therefore, translation into local languages may also have affected the findings [56], particularly where local languages may not have clear words to describe some of the questions related to psychosocial health problems. The study includes data collected over a 13-year period (2003–2015); therefore, the period effect could have biased the results. Furthermore, residual confounding variables (e.g., school-related factors such as school quality, school environment, learning difficulties, other mental health diagnoses, geography, and other cultural characteristics, etc.) could have influenced our findings as we were unable to adjust for such confounders due to variable limitations within the data set. Although the GSHS follows the complex sampling design, there are some effects of the cluster on independent and the dependent variables. Thus, to reduce cluster effect in the independent and the dependent variables multilevel is more efficient model. However, the authors could not able to perform multilevel model due to the paucity of require variables in these datasets; and this could be another limitation of this study.

## Conclusion

Our findings show that adolescents’ CSA remains a significant threat to social and health wellbeing of adolescents’ and can hinder education attainment. However, factors that accentuate CSA vary by cultural background, social economic status and gender. This calls for contextually appropriate, and gender-sensitive tailored interventions. The interventions should address the short term and long-term needs of the vulnerable adolescents and require creativity and flexibility to consider the varied circumstances (i.e., socio-economic, peer conflict, injury factors, and the risk of non-communicable diseases) of the affected adolescents. The short-term interventions should accelerate efforts to create safe spaces in schools and make them attractive for adolescents vulnerable to CSA. Additionally, the interventions should aim to stimulate and boost parental support, and involvement in their adolescent’s education. The long-term interventions require situating the construct CSA as a proxy for comprehensive interventions that address adverse and undesired lifelong consequences of food insecurity, violence and injury, victimisation, loneliness, anxiety and obesity. Such a double-barreled approach will ensure inclusivity and address both current and future needs and challenges associated with CSA.

### Ethics approval

We used publicly available data sources; hence, no additional ethical approval was required.

## Supporting information

S1 File Maps 1 & 2 show the prevalence of chronic school absenteeism (Boys and Girls) in the 1-months preceding survey completion among adolescents aged 11–17 years from low-middle-and-high-income countries, 2003–2015. (PDF)
