# Supplementary material for: Prevalence and factors associated with chronic school absenteeism among 207,107 in-school adolescents: Findings from cross-sectional studies in 71 low-middle and high-income countries
Source: PLoS One. 2023 May 10;18(5):e0283046. doi: 10.1371/journal.pone.0283046 (PMC10171665; doi:10.1371/journal.pone.0283046)
Supplement: S1 File — (PDF) [file pone.0283046.s001.pdf]

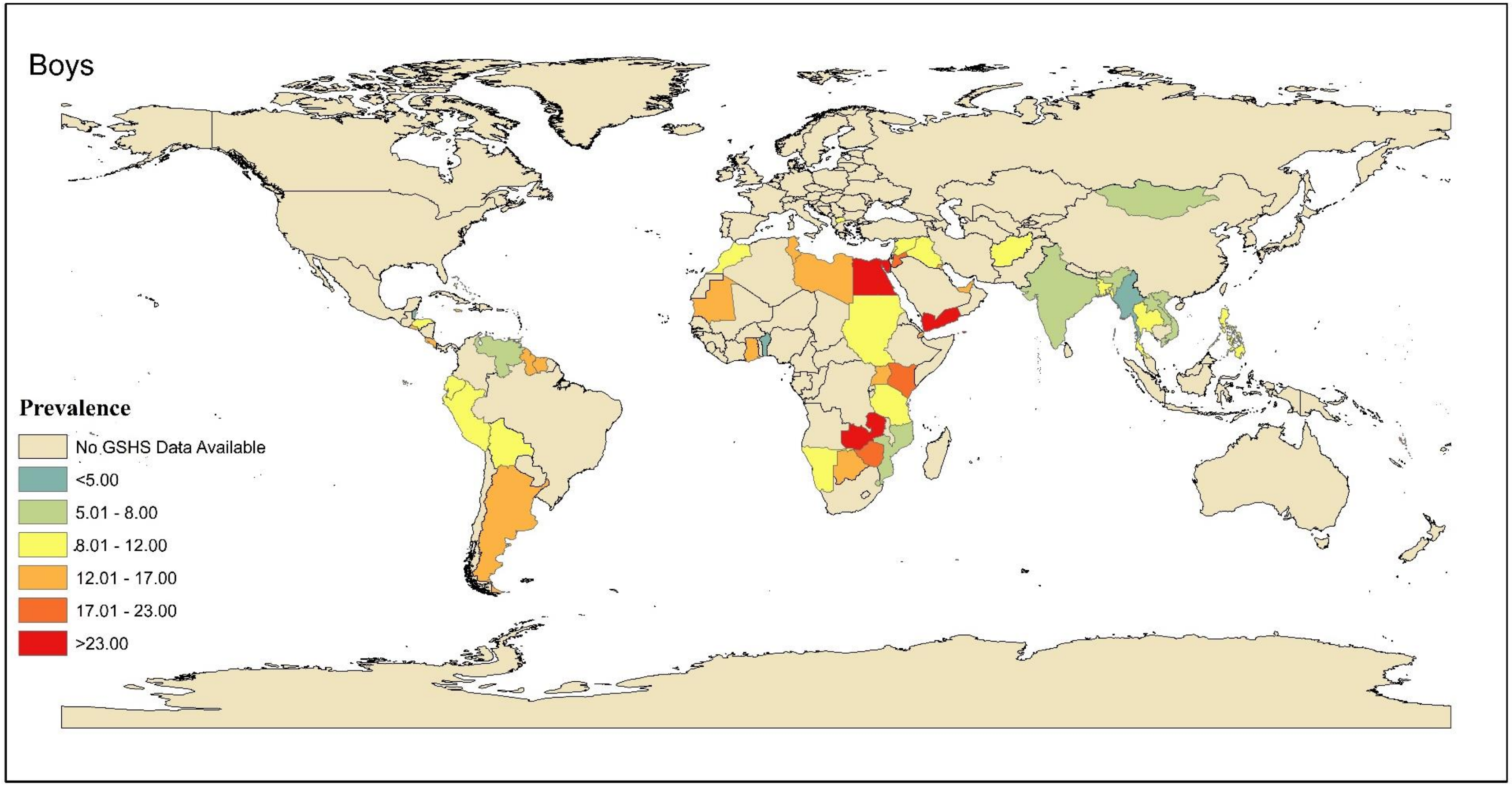

Supplementary Figure 1 Prevalence of Chronic School Absenteeism (Boys) in the 1–Months Preceding Survey Completion among Adolescents Aged 11–17 Years for 71 Low– Middle–and High–Income Countries, 2003–2015. A change in colour from green to red indicates a higher prevalence of chronic absenteeism.

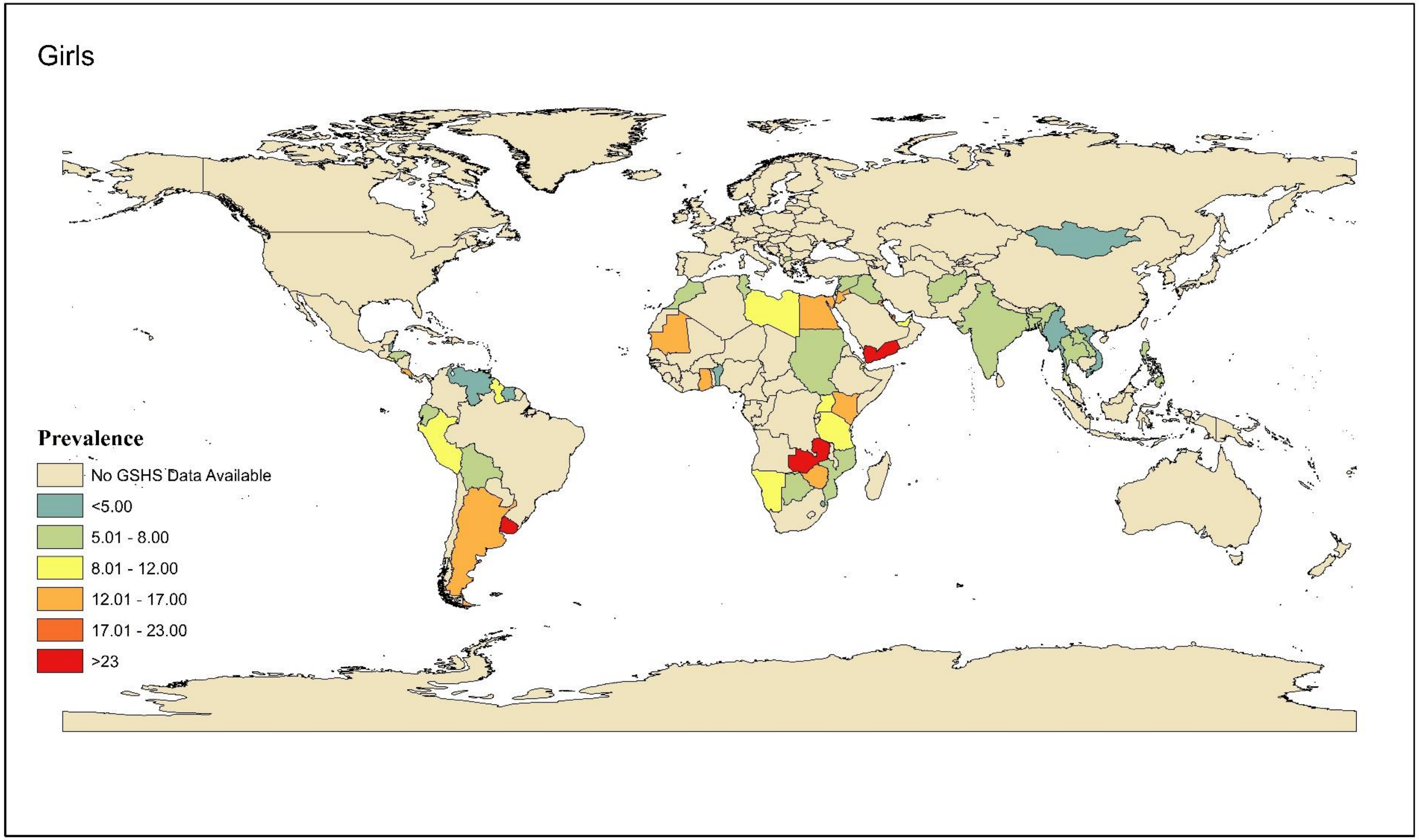

Supplementary Figure 2 Prevalence of Chronic School Absenteeism (Girls) in the 1–Months Preceding Survey Completion Among Adolescents Aged 11–17 Years for 71 Low– Middle–and High–Income Countries, 2003–2015. A change in colour from green to red indicates a higher prevalence of chronic absenteeism.
